# A prospective investigation into the association between the gut microbiome composition and cognitive performance among healthy young adults

**DOI:** 10.1186/s13099-022-00487-z

**Published:** 2022-04-19

**Authors:** Kolade Oluwagbemigun, Maike E. Schnermann, Matthias Schmid, John F. Cryan, Ute Nöthlings

**Affiliations:** 1 Nutritional Epidemiology, Department of Nutrition and Food Sciences, University of Bonn, Bonn, Germany; 2 Department of Medical Biometry, Informatics and Epidemiology, University Hospital Bonn, University of Bonn, Bonn, Germany; 3 APC Microbiome Ireland, University College Cork, Cork, Ireland

**Keywords:** Gut microbiome, Relative abundance, Ruminococcaceae, Coriobacteriaceae, Cognitive performance, Fluid intelligence, DONALD Study

## Abstract

**Background:**

There is emerging evidence that the gut microbiome composition is associated with several human health outcomes, which include cognitive performance. However, only a few prospective epidemiological studies exist and none among young adults. Here we address the gap in the literature by investigating whether the gut microbiome composition is prospectively linked to fluid intelligence among healthy young adults.

**Methods:**

Forty individuals (65% females, 26 years) from the DOrtmund Nutritional and Anthropometric Longitudinally Designed (DONALD) study provided a fecal sample for gut microbiome composition and subsequently (average of 166 days) completed a cognitive functioning test using the Cattell’s Culture Fair Intelligence Test, revised German version (CFT 20-R). The assessment of the gut microbiome at the genera level was by 16S rRNA V3-V4 Illumina sequencing. The relative abundance of 158 genera was summarized into bacterial communities using a novel data-driven dimension reduction, amalgamation. The fluid intelligence score was regressed on the relative abundance of the bacterial communities and adjusted for selected covariates.

**Results:**

The 158 genera were amalgamated into 12 amalgams (bacterial communities), which were composed of 18, 6, 10, 14, 8, 10, 16, 13, 12, 12, 3, and 11 genera. Only the 14-genera bacterial community, named the “Ruminococcaceae- and Coriobacteriaceae-dominant community” was positively associated with fluid intelligence score (β = 7.8; 95% CI: 0.62, 15.65, *P* = 0.04).

**Conclusion:**

Among healthy young adults, the abundance of a gut bacterial community was associated with fluid intelligence score. This study suggests that cognitive performance may potentially benefit from gut microbiome-based intervention.

## Background

It has become increasingly recognized that the gut microbiome may play a substantial role in the occurrence of many human conditions [1, 2]. More recently, the association of the gut microbiome with cognitive neurodevelopment and brain functioning has attracted much attention [3–7]. This association is attributed to the microbiota-gut-brain-axis [8, 9]. Indeed, epidemiological investigations have demonstrated associations between the composition of the gut microbiome and prevalent neurodegenerative conditions such as Alzheimer's disease [10–13] and Parkinson's disease [12, 14].

In apparently healthy individuals, the association between the gut microbiome composition and cognitive functioning is also documented [15–24]. However, these studies have yielded inconsistent results. These inconsistent results may reflect limitations of the design of these studies, which is mainly cross-sectional [15–17, 19–22, 24]. Therefore, more prospective studies where the gut microbiome composition is profiled before the assessment of cognitive performance will help to draw a more definite conclusion and contribute to improving our knowledge on the influence of the gut microbiome on cognitive functioning. Additionally, previous studies have been among infants [18, 23], children [24], middle-aged adults [20], and older adults [15–17, 19–22]. Considering that the cognitive health in young adulthood positively correlates with memory and brain functioning in later life [25], studies among young adults would be necessary.

Crystallized and fluid cognition (or intelligence) are the two cognitive domains [26]. The fluid intelligence is crucial because it is a person’s innate ability to process and learn new information, solve problems, and attend to and manipulate one’s environment [27]. In fact, it is positively associated with better psychological and health outcomes throughout adulthood and into old age [28]. Thus, the relationship between the gut microbiome and fluid intelligence should be of research interest.

To this end, the present prospective epidemiological study sought to investigate whether the gut microbiome composition is independently associated with fluid intelligence among young adults.

## Results

### Description of study population

Table 1 presents the basic characteristics of the 40 individuals in the current analysis. The median age at the time of fecal sampling was 26 years. About two-third (65%) were females. The median time between fecal sampling and the assessment of cognitive performance was 166 days. Furthermore, the birth weight was approximately 3.5 kg. Their BMI of 23.4 kg/m^2^ is within the normal weight range. They had a moderate physical activity of approximately 33 MET-hour/week. The median carbohydrate intake and alcohol consumption were 199 g/day and 0.39 g/day, respectively. The median Shannon alpha diversity of the gut microbiome was 6.1. Finally, the median fluid intelligence score was 110.

**Table 1:** Basic characteristic of the study population (N = 40)

	N	
Sex, females^a^	40	26 (65)
Age, years^b^	40	26 (22, 30)
Birth weight^b^	40	3480 (3215, 3665)
Body mass index, kg/m^2b^	40	23.43 (21.08, 25.06)
Physical activity, MET-hour/week^b^	40	33.14 (21.72, 51.12)
Energy intake, kcal/day^b^	40	1551.3 (1418.96, 1746.72)
Carbohydrate intake, g/day^b^	40	198.62 (173.97, 230.34)
Fiber intake, g/day^b^	40	14.69 (13.10, 16.63)
Protein intake, g/day^b^	40	51.02 (46.87, 56.87)
Fat intake, g/day^b^	40	61.17 (55.61, 67.5)
Alcohol consumption, g/day^b^	40	0.39 (0.21, 1.1)
Smoking status, current smokers^a^	37	6 (16)
Education, ≥ 12 years of education^a^	40	22 (55)
Antibiotics intake^a^	33	13 (39)
Probiotics intake^a^	33	17 (52)
Time between fecal sampling microbiome and cognition assessment, days^b^	40	166 (130, 194.5)
Shannon alpha diversity index^b^	40	6.1 (5.86, 6.3)
Fluid intelligence score^b^	40	110 (100, 119)

^a^n(%)

^b^Median (25th, 75th percentile)

n = count, % = percentage

Table 2 shows the 133 assigned genera and the 12 mutually exclusive amalgams to which they were assigned. These 12 amalgams were composed of 18, 6, 10, 14, 8, 10, 16, 13, 12, 12, 3, and 11 genera, respectively. They include well-known genera such as *Fusobacterium*, *Lachnoclostridium*, *Staphylococcus*, *Akkermansia*, *Bacteroides*, *Streptococcus*, *Phascolarctobacterium and Desulfovibrio*, *Enterococcus*, *Paraprevotella*, *Blautia*, *Pseudobutyrivibrio*, and *Dialister*, respectively. Twenty-five genera were not assigned.

**Table 2:** The twelve amalgams of the gut microbiome and their description

Amalgams	Numbers of genera	Genera	Description
V1	18	*Tyzzerella, Lachnospiraceae FCS020 group, Howardell, Prevotellaceae NK3B31 group, Alloprevotella*, *Coprobacillus, Intestinibacter, NB1-n uncultured bacterium, Family XIII AD3011 group, Prevotella 7***,** *Uncultured Thermoanaerobacterales bacterium, Hafnia, Eubacterium*, *Bacteroidales S24-7 group uncultured organism, Lactobacillus, Fusobacterium*, *Veillonellaceae uncultured bacterium, Peptococcus*	Lachnospiraceae-dominant community I
V2	6	*Lachnospiraceae FE2018 group***,** *Lachnoclostridium, Ruminococcus 2, Ruminococcaceae UCG-009, Erysipelotrichaceae UCG-003, Defluviitaleaceae UCG-011*	Lachnospiraceae- and Ruminococcaceae-dominant community
V3	10	*Lachnospiraceae NC2004 group, Tyzzerella 4*, *R-7 group, Christensenellaceae uncultured bacterium*, *Clostridium *sensu stricto* 1, Veillonella*, *Anaerotruncus*, *Staphylococcus, Mollicutes RF9 uncultured bacterium*, *Pediococcus*	Lachnospiraceae- and Christensenellaceae-dominant community
V4	14	*Ruminiclostridium 5, Ruminococcaceae UCG-010*, *Coriobacteriaceae uncultured bacterium, Slackia*, *[Eubacterium] hallii group*, *Peptoclostridium*, *Akkermansia*, *Lactococcus*, Erysipelotrichaceae *Incertae Sedis*, *[Eubacterium] nodatum group*, *Prevotellaceae uncultured bacterium*, *Robiginitalea*, *Pseudomonas*, *Bacteroidales S24-7 group uncultured bacterium*	Ruminococcaceae- and Coriobacteriaceae-dominant community
V5	8	*Erysipelotrichaceae bacterium 21_3, Erysipelotrichaceae UCG-004*, *Bacteroides, Anaerostipes*, *Clostridiales vadinBB60 group uncultured organism*, *Coprobacter, Megasphaera*, *boneC3G7 uncultured bacterium*	Erysipelotrichaceae-dominant community I
V6	10	*Roseburia, Lachnoclostridium 5, Hungatella***,** *Streptococcus, Terrisporobacter*, *Oscillospira***,** *Uncultured Mollicutes bacterium*, *Acidaminococcus*, *Haemophilus*, *Acinetobacter*	Lachnospiraceae-dominant community II
V7	16	*Turicibacter, Holdemanella, Catenibacterium, Ruminococcaceae UCG-002, Flavonifractor, Allisonella, Megamonas*, *Gastranaerophilale uncultured bacterium, Escherichia-Shigella, Eggerthella, Phascolarctobacterium, Clostridiales vadinBB60 group uncultured bacterium, Lachnospiraceae uncultured bacterium*, *Prevotellaceae UCG-003*, *Desulfovibrio, Anaeroplasma*	Erysipelotrichaceae-dominant community II
V8	13	*Fusicatenibacter, Tyzzerella 3, Eisenbergiella, Ruminococcaceae UCG-013, Butyricicoccus*, *Parasutterella, Sutterella*, *Erysipelatoclostridium, Solobacterium*, *Prevotellaceae UCG-001, Enterococcus*, *Thalassospira, Enterorhabdus*	Lachnospiraceae-dominant community III
V9	12	*Intestinimonas, Ruminiclostridium 6, Ruminiclostridium 1, Lachnospiraceae NK4A136 group, Lachnospiraceae UCG-001*, *Butyricimonas*, *Rikenellaceae RC9 gut group, Senegalimassilia***,** *Raoultella, NB1-n uncultured organism*, *Mitsuokella, Paraprevotella*	Ruminococcaceae-dominant community I
V10	12	*Faecalibacterium, Ruminococcaceae UCG-004, Ruminiclostridium, Ruminococcaceae UCG-009, Ruminiclostridium 9*, *Blautia, [Eubacterium] ventriosum group*, *Bilophila, Erysipelotrichaceae UCG-003, Defluviitaleaceae UCG-011*, *Prevotella 9*, *Collinsella*	Ruminococcaceae-dominant community II
V11	3	*Pseudobutyrivibrio, [Ruminococcus] gauvreauii group, [Eubacterium] ruminantium group*	Lachnospiraceae community
V12	11	*Odoribacter, Porphyromonadaceae uncultured, Barnesiella*, *Subdoligranulum, Ruminococcaceae UCG-003*, *Asteroleplasma, Methanobrevibacter, Opitutae vadinHA64 uncultured bacterium*, *Lachnospiraceae UCG-003***,** *Dialister*, *Alistipes*	Porphyromonadaceae-dominant community
V0	25	*Lachnospiraceae UCG-004, Lachnospiraceae UCG-005, Lachnospiraceae UCG-008, Dorea, Coprococcus 1, Coprococcus 2, Lachnospiraceae, uncultured, Lachnospira, Marvinbryantia, Shuttleworthia, Ruminococcaceae NK4A214 group, Ruminococcaceae UCG-005, Ruminococcaceae UCG-014, [Eubacterium] coprostanoligenes group, Ruminococcaceae uncultured bacterium, Ruminococcus 1, Rhodococcus*, *Bifidobacterium, Parabacteroides*, *Faecalitalea*, *Victivallis*, *Cloacibacillus, Enterobacter*, *Prevotella 2*, *Succiniclasticum*	Group of unassigned genera

**Fig. 1:**
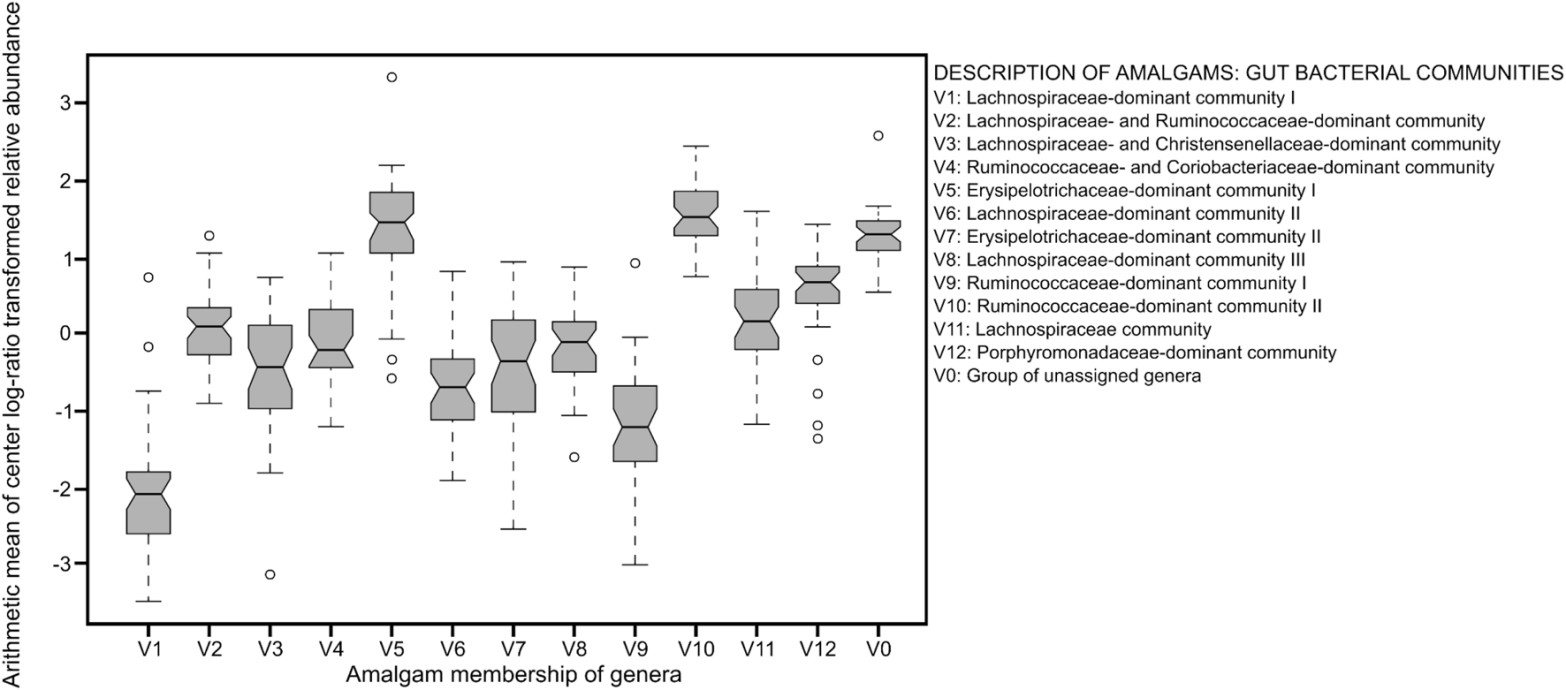
Boxplots for the average center log-ratio transformed relative abundance of each amalgam

The boxplots for the average center log-ratio transformed relative abundance (RA) of all genera in each amalgam (Fig. 1) shows that the lowly abundant genera were grouped together in one amalgam. The inter-quartile range of ten amalgams being lower than the unassigned group also suggests that lowly abundant genera were not systematically excluded by amalgamation. Further, the fact that the 12 amalgams and the unassigned group overlapped with at least one other group suggests that none of the groups was different from all others. The 12 amalgams were named as follows: “Lachnospiraceae-dominant community I", “Lachnospiraceae- and Ruminococcaceae-dominant community”, “Lachnospiraceae- and Christensenellaceae-dominant community”, “Ruminococcaceae- and Coriobacteriaceae-dominant community”, “Erysipelotrichaceae-dominant community I”, “Lachnospiraceae-dominant community II”, “Erysipelotrichaceae-dominant community II”, “Lachnospiraceae-dominant community III”, “Ruminococcaceae-dominant community I”, “Ruminococcaceae-dominant community II”, “Lachnospiraceae community”, and “ Porphyromonadaceae-dominant community”. Further, among the bacterial communities, there were no exceptionally high pairwise correlations with the maximum correlation being moderate (|0.5|≤ r ≤|0.7|, *P* < 0.05) and appearing only in eight correlations. The Shapiro–Wilk W test for normality (*P* = 0.51) on fluid intelligence score indicated that it has a normal distribution with constant variance. Thus, fluid intelligence score was modelled untransformed.

**Fig. 2:**
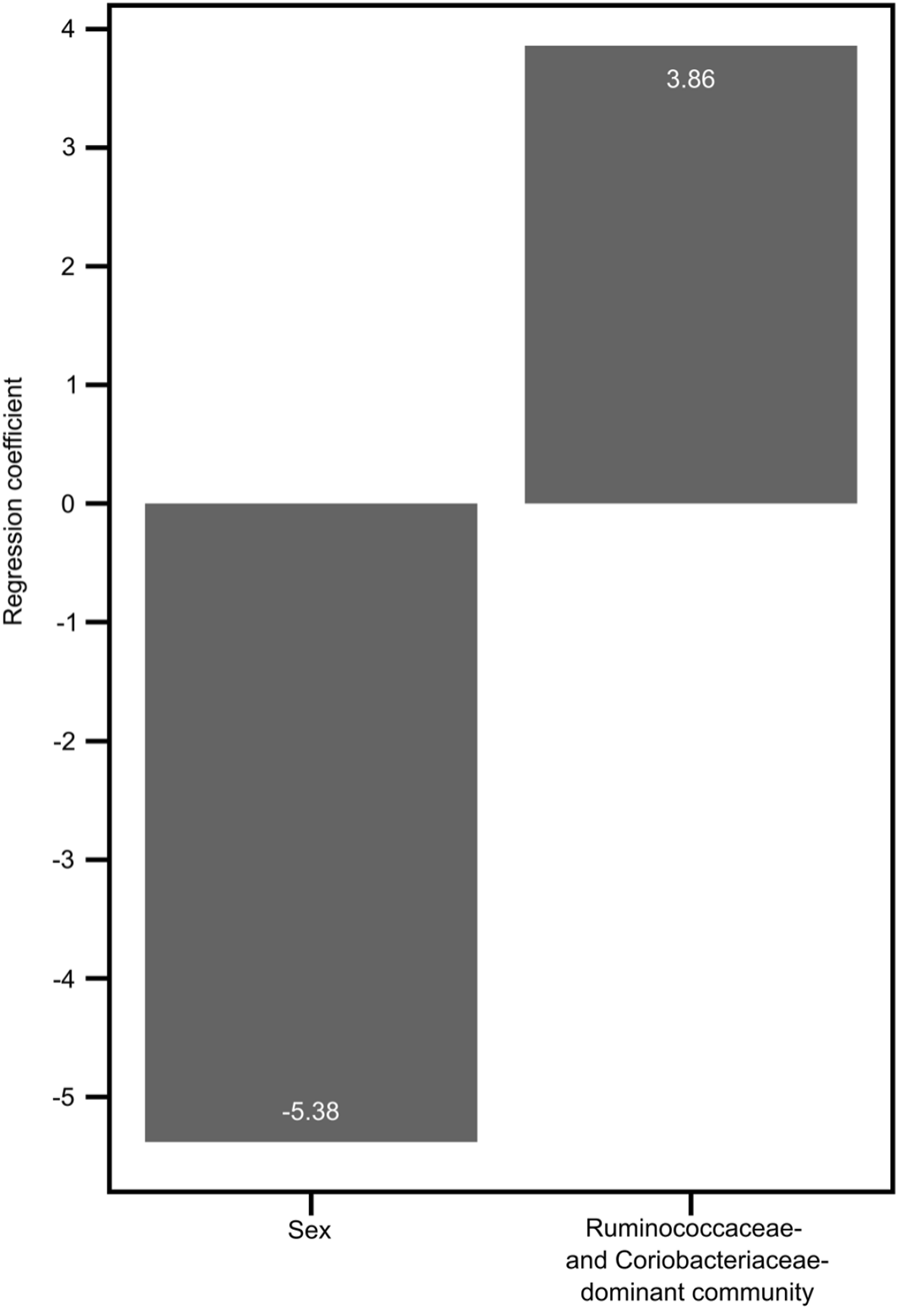
The non-zero predictors of fluid intelligence score obtained from the adaptive LASSO regression

In the adaptive LASSO, the value of the lambda that gives the minimum mean cross-validated error was 84.48. There were two predictors of fluid intelligence score with non-zero coefficients at this value. Figure 2 displays these two predictors, the “Ruminococcaceae- and Coriobacteriaceae-dominant community” and sex, with their regularized regression coefficients of 3.86 and − 5.38, respectively.

**Fig. 3:**
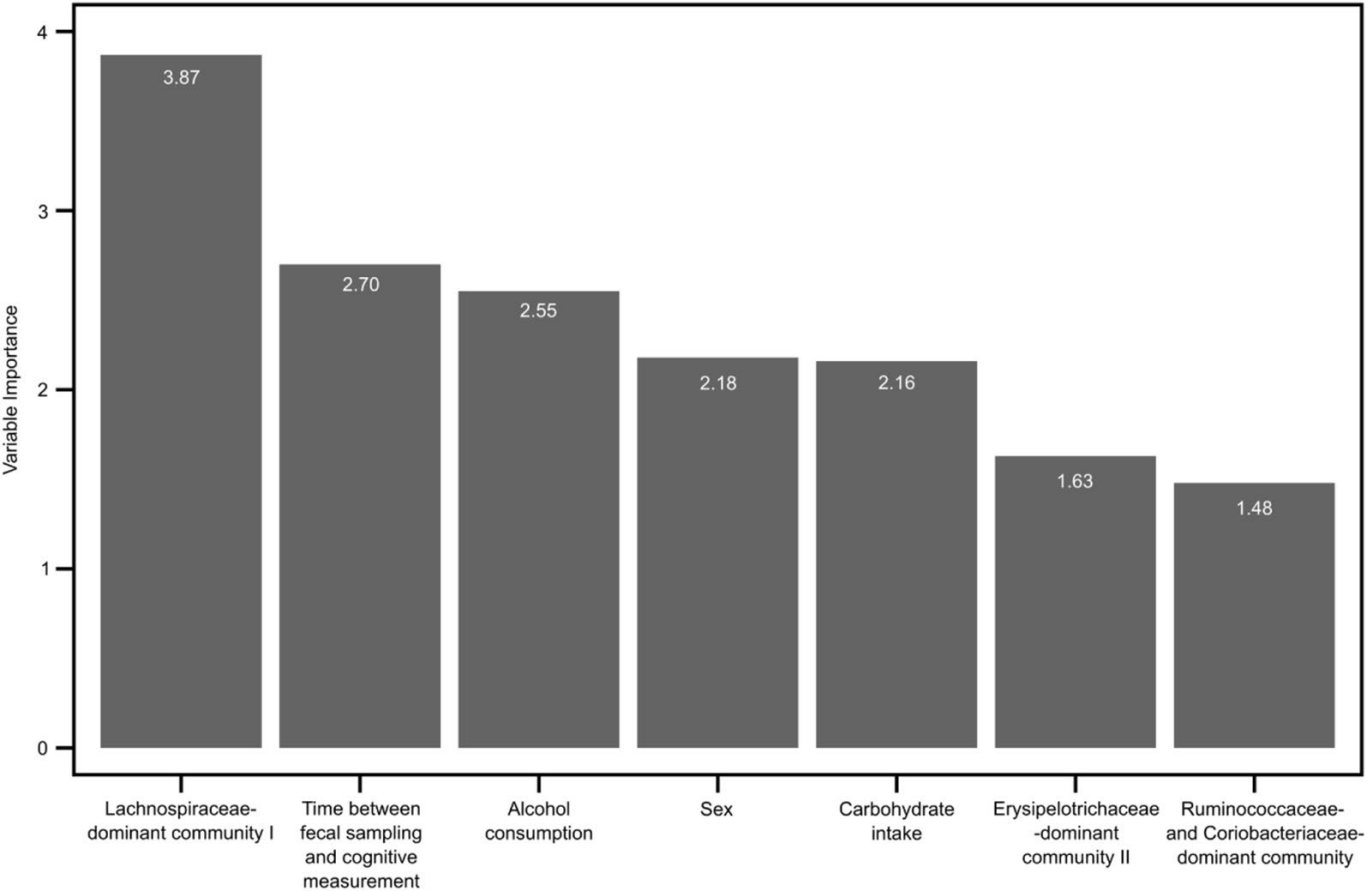
The seven optimal predictors of fluid intelligence score obtained from the random forest regression with recursive feature elimination

Additionally, the RF-RFE recommends that seven predictors of fluid intelligence score were optimal when the root-mean-square error, R-squared, and the mean absolute error reached their maximum levels at 13.18, 0.07, and 10.76, respectively. These seven predictors were “Lachnospiraceae-dominant community I”, time between fecal sampling and cognitive measurement, alcohol consumption, sex, carbohydrate intake, “Erysipelotrichaceae-dominant community II”, and “Ruminococcaceae- and Coriobacteriaceae-dominant community” with importance scores of 3.87, 2.7, 2.55, 2.18, 2.16, 1.63, and 1.48, respectively (Fig. 3). Thus, the true relevant predictors of fluid intelligence score predictors, shared by the adaptive LASSO and RF-RFE, were Ruminococcaceae- and Coriobacteriaceae-dominant community and sex.

Regressing fluid intelligence score on “Ruminococcaceae- and Coriobacteriaceae-dominant community” and sex using the ordinary least squares regression showed that “Ruminococcaceae- and Coriobacteriaceae-dominant community” was positively associated with fluid intelligence score (β = 7.8; 95% CI: 0.62, 15.65, *P* = 0.04) and females had lower intelligence score when compared to males (β = − 9; 95% CI: − 17.36, − 0.71, *P* = 0.03). The model F statistic was significant (F = 4.05, *P* = 0.03), indicating that the model accounts for a significant portion of the variation in the data. The adjusted R-squared indicates that these two predictors explained approximately 14% of the variation in fluid intelligence score.

## Discussion

The current prospective epidemiological study among young adults investigated whether the gut microbiome composition was independently associated with fluid intelligence. Among the 12 bacterial communities retrieved from this study population, the “Ruminococcaceae- and Coriobacteriaceae-dominant community” was positive and independently associated with fluid intelligence. This 14-genera community comprises *Ruminiclostridium 5*, *Ruminococcaceae UCG-010, Coriobacteriaceae uncultured bacterium*, *Slackia*, *[Eubacterium] hallii group*, *Peptoclostridium, Akkermansia*, *Lactococcus*, *Erysipelotrichaceae incertae sedis*, *[Eubacterium] nodatum group*, *Prevotellaceae uncultured bacterium*, *Robiginitalea*, *Pseudomonas*, and, *Bacteroidales S24-7 group uncultured bacterium*.

Our finding echoes those of previous epidemiological investigations, which reported the relationship of Ruminococcaceae and Coriobacteriaceae with cognitive functioning. The abundance of Ruminococcaceae was positively associated with good cognition [15, 29]. A study with a probiotic supplementation showed that an increase in the abundance of a genus in Ruminococcaceae resulted in better protection against the negative effects of stress on working memory [30]. Furthermore, it was reported that the abundance of Coriobacteriaceae was positively associated with a better cognitive performance [20]. The abundance of Ruminococcaceae was reduced in Alzheimer disease [29], multiple sclerosis [31] and schizophrenia [32], and the abundance of many of its species was reduced in Parkinson's disease [14]. Some of the genera in this community are also associated with cognition. The abundance of *Erysipelotrichaceae incertae sedis* and *[Eubacterium] hallii group* were positively associated with cognitive functioning [15] and social cognition [33], respectively. Besides, two intervention studies reported that the ingestion of multispecies probiotics, which includes *Lactococcus*, was associated with reduced cognitive reactivity to mood disorders [34, 35]. Diet-induced increase in the abundance of *Akkermansia* and *Slackia* was associated with improved Alzheimer's disease biomarkers in individuals with mild cognitive impairment [36]. There was also a reduced abundance of *Slackia* in multiple sclerosis [37]. The fact that the majority of these aforementioned results are in a similar direction as ours suggests that our findings are reliable and biologically relevant.

The first potential biological mechanism by which the gut microbiome influence cognitive functioning is through its stimulation of the afferent neurons of the enteric nervous system that communicates with the central nervous system via the vagus nerve [9]. In addition, the gut microbiome possesses the ability to produce and modify various immune, metabolic, and neuroactive factors that affect the central nervous system [9]. Important neuroactive factors are produced from the gut microbiome’s modulation of the dietary protein and carbohydrate metabolism [9]. First, *Lactococcus* [38] and *Pseudomonas* [39] are able to modulate the serotonin signaling/metabolism. *Akkermansia* was predicted to be able to produce serotonin [40]. Further, *Pseudomonas* is one of the gamma aminobutyric acid-modifying genera [41] and gamma aminobutyric acid level is lower in individuals with Alzheimer's disease as compared to healthy individuals, [42]. *Lactococcus* produces dopamine [43] and histamine [44, 45] that regulate cognitive functions. *Akkermansia* and *[Eubacterium] hallii group* produce short chain fatty acids (SCFA) from carbohydrate metabolism. *Akkermansia* produces acetate and propionate [46], while *[Eubacterium] hallii group* produces propionate [2] and butyrate [10]. The production of these SCFA generally has a beneficial influence on many neurodegenerative conditions [10]. Indeed, systemic acetate has the capability to cross the blood–brain barrier where it can activate acetyl-CoA carboxylase leading to the enhancement of the expression of neuropeptides that induces hypothalamic neuronal activation and suppresses appetite [47]. *Akkermansia* also tend to produce indole and indole acetic acid from tryptophan metabolism [39]. The indirect mechanism through inflammation implicates *Akkermansia* and *Slackia* [48–50]. *Akkermansia* plays a critical role in maintaining the integrity of the mucin layer and reducing inflammation [48]. *Slackia* is an equol producer [49] and equol is crucial in maintaining immune homeostasis because it induces anti-inflammatory response [50]. The SCFA also act as anti-inflammatory mediators [51]. Clearly, these mechanisms may critically interact with one another in complex ways. Considering that only a limited number of genera in this community has documented potential mechanism of action, further work is a needed for insights into how the bacteria in this community work together to impact cognition.

The two prospective studies, which are both among infants, reported that Firmicutes-dominant and Bacteroidetes-dominant clusters [23], *Bacteroides*-dominant cluster [18], and *Bacteroides* [23] were positively associated with a favorable cognitive function. The *Bacteroides*-dominant cluster of Carlson et al. [18] and our “Erysipelotrichaceae-dominant community I” both have *Bacteroides*. Thus, they are comparable. The fact that the “Erysipelotrichaceae-dominant community I” and “Lachnospiraceae-dominant community I” with the highest and lowest variance respectively were not associated with fluid intelligence suggests that the absence of association of “Erysipelotrichaceae-dominant community I” with fluid intelligence is unlikely to be influenced by its variation. Therefore, our finding for the “Ruminococcaceae- and Coriobacteriaceae-dominant community” suggests that the relationship between the gut microbiome composition and cognitive function may be different between infants and young adults. Furthermore, between three to five genera in the “Ruminococcaceae- and Coriobacteriaceae-dominant community” consistently cluster together across all the tested number of amalgams. This suggests that the membership of this community is not arbitrary but highly reproducible and the community might indeed represent a relevant biomarker. Furthermore, the bacterial communities retrieved in this study are consistent with other studies using different dimension reduction methods. A study among older German adults was also able to recover a Ruminococcaceae-dominated bacterial community and a community including the Coriobacteriaceae [52]. The *[Eubacterium] hallii group* and *Peptoclostridium* in our “Ruminococcaceae- and Coriobacteriaceae-dominant community” were also among the genera in one of the bacterial communities of Leong et al. [53]. Besides, our “Erysipelotrichaceae-dominant community I” and “Ruminococcaceae-dominant community II” are somewhat comparable to the *Bacteroides*- and *Faecalibacterium-*dominant clusters of Carlson et al. [18].

A recent study in a different population showed that age, sex, education, average food intake, and tyrosine intake explained 6% of the variance of fluid intelligence [54]. This is lower than the 14% variance of fluid intelligence explained by the abundance of the “Ruminococcaceae- and Coriobacteriaceae-dominant community” and sex in our study. This finding underscores the importance of the gut microbiome composition in fluid intelligence. Sex disparity in different aspects of cognitive performance is well documented and much debated [55–58]. In consort with our findings, studies among young adults that assessed fluid intelligence by the CFT 20-R [56] and the Wechsler Adult Intelligence Scale [58] reported that males have a higher score than females. Moreover, considering that our previously reported core genera and the carbohydrate intake-related genera [59] were not part of this fluid intelligence-associated bacterial community suggests that there might be a minimal association between the core gut microbiome and carbohydrate intake-induced changes in the gut microbiome abundance in this study population and their cognitive performance. Nonetheless, this does not exclude that diet or other lifestyle factors may induce changes in the abundance of this gut bacterial community.

There are some limitations of the present study; therefore, our findings should be interpreted with a degree of caution. This is an observational study; hence, it cannot provide a definitive conclusion regarding cause and effect. Whilst we adjusted for important factors that might influence the gut microbiome abundance and cognitive performance, unmeasured factors such as genetics, socioeconomic status, stool consistency, and measures of brain health/brain structure and function or imprecisely measured factors could have resulted in residual confounding. Furthermore, despite the acceptable power of this study sample, the confidence interval of our effect estimate is wide. Additionally, we did not include interactions between our predictors in our statistical model because there is no sufficient prior research on specific interactions among the predictors with respect to cognition. However, our result suggests that sex-specific relationship between the gut microbiome abundance and cognitive performance should be considered in other studies. In addition, we did not measure cognitive performance as broadly or deeply as some of the previous studies. However, it was demonstrated that fluid intelligence correlates with other measures of cognitive performance [60]. Therefore, it is likely that our findings would be similar for other measures of cognitive performance. Nevertheless, future studies with a larger study population should incorporate broader and deeper phenotyping of cognitive performance such as functional brain networks. The current study considered the gut microbiome composition; thus, it was impossible to determine how much the gut microbiome-derived metabolites and inflammatory markers could have mediated or modified the association between the gut microbiome composition and fluid intelligence. Other studies should consider the longitudinal assessment of gut microbiome composition, gut microbiome-derived metabolites, inflammatory markers, and cognitive performance in design and analysis. Despite taking the time between fecal sampling and cognitive measurement into account in our analysis, it is possible that our findings could have been different for different average follow-up times. The convectional analytical approach for differential abundance is to first test for an effect among samples with the variable of interest, and if there is a significant difference among samples, to then test for what features are driving the effect using methods such as Permutational multivariate analysis of variance or Adonis. However, the starting point of the current analysis was to capture the inter-taxa relationship among genera, to model our cognition performance (fluid intelligence score) as the outcome variable, which is supported by the direction of the biological relationship between gut microbiome and cognition and our prospective study design, and to model the fluid intelligence score without categorization. All these aims would have been difficult to achieve simultaneously using the convectional analytical approach. Finally, bias in any step of our microbiomics workflow such as fecal sample collection and preservation, DNA extraction, library preparation, sequencing, or bioinformatics, could have influenced our results.

A major strength of the current work is that it is a prospective observational study, in which the assessment of the exposure, gut microbiome composition precedes the outcome, fluid intelligence. Thus, our study design offers a reliable evidence of the association between the gut microbiome composition and fluid intelligence. To the best of our knowledge, this is also the first epidemiological study to report on the association between the gut microbiome composition and fluid intelligence among young adults. Other strengths are that we performed a priori power analysis and our statistical model building was theory driven. We also conducted complementary regression analyses to ensure the robustness of our results. Furthermore, our study population is young adults; therefore, the impact of reverse causality is likely low. Thus, it is unlikely that we could have overestimated the true association between gut microbiome composition and fluid intelligence score. Nevertheless, better-designed larger prospective studies among young adults are needed to confirm our findings.

## Conclusion

This study provides an interesting finding that the abundance of 14 interacting genera in the gut microbiome is positively linked to fluid intelligence score. This lends credence to the growing evidence that the gut microbiome may influence cognitive performance. Taken together, our study suggests that cognitive performance may potentially benefit from gut microbiome-based intervention and this group of bacteria may have a promising health-promoting role.

## Methods

### Study population

The DOrtmund Nutritional and Anthropometric Longitudinally Designed (DONALD) study is an ongoing, open prospective epidemiologic cohort study of individuals living in the German town of Dortmund and surrounding cities that commenced in 1985. The study was designed to investigate the relationships among dietary intake, metabolism, and growth from infancy into adulthood. Participants’ examinations included annually repeated anthropometric measurements and three-day weighed dietary records. Early-life factors such as birth weight of study participants were extracted from maternal delivery documents. The study was conducted in accordance with the Declaration of Helsinki and was approved by the Ethics Committee of the University of Bonn. Informed consent was obtained from the parents or legal guardians of the participants in childhood and later on from the participants themselves. Details of the recruitment and follow-up in the DONALD study are presented elsewhere [61].

### Study design

This is a prospective study of adult (age ≥ 18 years) DONALD study participants who provided fecal samples for gut microbiome compositional analysis [59] and who subsequently attended cognitive testing few months after fecal sampling. These individuals were singletons, full term (36–42 weeks) and birth weight of ≥ 2500 g. There were 40 individuals in total. For these 40 individuals, we retrieved their gut microbiome RA data, cognition data, and other covariates.

### Assessment of gut microbiome composition

Details of the fecal sampling, DNA extraction, 16S ribosomal RNA sequencing have been published [59]. Briefly, fecal samples were collected between 2017 and 2018 at the participants’ home into tubes containing *RNAlater* (Qiagen) and sent to the Biobank within 24 h of collection. Bacterial genomic DNA was extracted from 0.25 g of fecal sample using the repeat bead beating plus column protocol as in combination with the QIAamp Fast DNA Stool Mini Kit. For the 16S ribosomal RNA sequencing, the V3-V4 regions of the 16S rRNA gene were amplified through 30 cycles of PCR reactions according to the 16S Metagenomic Sequencing preparation protocol for Illumina MiSeq. The Quantitative Insights into Microbial Ecology (QIIME version 1.8.0) was used for quality filtering of pair-end reads, which is based on a quality score of > 25 and the removal of mismatching barcodes. Prior to the analysis as part of the quality control process, sequences which produced less than 40,000 reads were manually removed. USEARCH (version 7, 64-bit) was employed for deionization, chimera detection and clustering into operational taxonomic units (OTUs; 97% identity). Alignment of OTUs was carried out using the python nearest alignment space termination (PyNAST) and assignment of taxonomy of 97% similar identity against the SILVA SSURef database release v123. The R package Phyloseq was used to determine 341 genera and their relative abundances. The 158 abundant genera with RA ≥ 0.2% in at least 10% of the samples [59] were considered for this present study.

### Assessment of cognitive performance, fluid intelligence

The cognitive performance was assessed between 2017 and 2018 using the Cattell’s Culture Fair Intelligence Test, revised German version (CFT 20-R) [62]. This computer-based CFT 20-R was done in the participants’ homes. The test comprises two parts, covering four domains: series, classifications, matrices, and topologies. The first and second parts comprised 56 and 45 figure-based questions and are to be complete within 14 min (4-4-3-3 min) and 12 min (3-3-3-3 min), respectively. The sum score of the domains was calculated from the correct answers, separately for each part. Age-standardized intelligence quotient (IQ) scores were derived from these sum scores to form IQ1 for the first part, IQ2 for the second part, and overall IQ–average of IQ1 and IQ2. If there was a difference ≥ 12 points between IQ1 and IQ2, the higher IQ was chosen as fluid intelligence score, otherwise the overall IQ was chosen as the fluid intelligence score.

### Assessment of other covariates

Demographic information such as sex and birth weight were retrieved from maternal delivery records. Age at fecal sampling was calculated from the documented date of birth and the date of fecal sampling. Dietary intake was assessed annually using three-day weighed dietary records on three consecutive days. From all dietary records prior to fecal sampling (age ≤ 18 years), means of daily energy (kcal/day) and carbohydrate, fiber, protein, and fat (g/day) intakes were calculated. Anthropometric measurements were conducted annually at the study center. For the current study, we considered the weight and height measurement closest to fecal sampling to calculate the body mass index (BMI). Physical activity was self-reported in a validated questionnaire covering the frequency and duration of individuals’ participation in the home and leisure physical activities during the week and at weekends. This was converted to metabolic equivalent of task (MET)-hours/week and the average over the available records of age ≤ 18 years was calculated for each individual. Educational status and lifestyle factors of the participants such as alcohol consumption and smoking, and self-reported intake of antibiotics and probiotics six months prior to fecal sampling, were obtained by questionnaires.

### Statistical analyses

Continuous and categorical variables were summarized as median (25% and 75% percentile), and as count (percentage), respectively.

Microbiome datasets are typically compositional, high dimensional, and zero-inflated. Hence, modeling its individual variables (taxa), particularly as predictors in convectional regression models is suboptimal [63]. The conventional and appealing statistical methods that efficiently model microbiome data include the simple log-ratio, log-ratio between two geometric means (balances), and summed log-ratio with the summed log-ratio being arguably the most interpretable [64]. Recently, a data-driven summed log-ratio method called amalgamation for reducing the dimensionality of compositional data has been proposed. [64]. Amalgamation outperforms traditional dimension reduction approaches, especially in terms of interpretability [64]. The resulting components (amalgams) from amalgamation can be described as bacterial communities [64].

For this current analysis, we considered the previously reported 158 abundant genera in this study population [59]. These include the core genera, *Bacteroides*, *Lachnoclostridium*, and *Blautia*, and the diet-related genera, *Phascolarctobacterium*, *Dialister*, and *Desulfovibrio* [59]. The zero counts in the abundance of the genera were replaced by the Bayesian-multiplicative method. Afterwards, we applied the amalgamation method. We started with three amalgams, using the simplest amalgamation where each genus only contributes to one amalgam and an objective function that preserves the Aitchison distances between samples. The number of amalgams was increased by one until we achieved amalgams where *Bacteroides*, *Lachnoclostridium*, *Blautia*, *Phascolarctobacterium*, *Dialister*, and *Desulfovibrio* were loaded. This was achieved at 12 amalgams. The amalgams (bacterial communities) were named according to the dominating bacterial family. As recommended by Quinn et al. [64], we center log-ratio-transformed the resultantly assigned amalgams for further analysis.

A minimum of approximately two observations per variable is adequate for a valid linear regression model [65]. Therefore, for our 40 observations, we will require a maximum of 20 predictors. This implies that our model should comprise the 12 bacterial communities and a maximum of eight covariates. Additionally, we estimated the power of our multivariable linear regression with 40 observations (n = 40), 20 predictors, a significance level of 0.05, and 46% average variation in cognitive performance captured by models comprising age, sex, and RA of the gut microbiome reported by another study [20]. With these values, the power of our multivariable linear regression model would be 72%, which is close to the convectional threshold of 80% [66]. We selected covariates, such as age [67, 68], sex [68, 69], BMI [70, 71], carbohydrate intake [59, 72], and alcohol consumption [73, 74] for which the direction of association with gut microbiome composition and cognition is well substantiated in the literature. We used these covariates to draw directed acyclic graphs in order to determine the minimal sufficient adjustment sets for estimating the direct effect of the gut microbiome RA on cognition. From these covariates, we selected eight covariates in the study population that were measured and have no missing. These were sex (reference: female), age at fecal sampling, BMI at fecal sampling, average physical activity, Shannon alpha diversity index, average carbohydrate intake, average alcohol consumption, and time between fecal sampling and cognitive measurement. In addition, we assessed whether the response variable, fluid intelligence score has a normal distribution with constant variance using the Shapiro–Wilk W test for normality.

Furthermore, using two variable selection regression methods, the adaptive Least Absolute Shrinkage and Selection Operator (LASSO) regression and the random forest regression with recursive feature elimination (RF-RFE), we identified true relevant predictors of fluid intelligence score by regressing it on the center log-ratio-transformed amalgams and the selected covariates. For both methods, we used five-fold cross-validation. The coefficient-specific penalty level of the LASSO was the inverse of the absolute values of the best ridge coefficients. We extracted the predictors with non-zero coefficients at the value of the lambda that gives the minimum mean cross-validated error. In the RF-RFE, we selected the optimal set of predictors when the root-mean-square error, R-squared, and the mean absolute error reached the maximum level. The true relevant predictors were the predictors shared by adaptive LASSO and RF-RFE. Finally, in order to obtain unbiased estimates for the true relevant predictors, we regressed fluid intelligence score on them using ordinary least squares regression using a five-fold cross-validation.

All statistical analyses were performed using the statistical software R version 4.1.1. The replacement of zeros, amalgamation, the adaptive LASSO, and the RF-RFE were implemented using the cmultRepl function from the zCompositions package, the amalgam package, the glmnet package, and the caret package, respectively.

## Data Availability

The datasets generated and/or analyzed during the current study are not publicly available due ethical and privacy issues, but they are available from the corresponding author on reasonable request and the approval of the principal investigator.

## Abbreviations

BMI: Body mass index
CFT 20-R: Cattell’s Culture Fair Intelligence Test, revised German version
DONALD: DOrtmund Nutritional and Anthropometric Longitudinally Designed
IQ: Intelligence quotient
LASSO: Least Absolute Shrinkage and Selection Operator
MET: Metabolic equivalent of task
RF-RFE: Random forest regression with recursive feature elimination
RA: Relative abundance
SCFA: Short chain fatty acids
